# Predictions of Mortality from Pleural Mesothelioma in Italy After the Ban of Asbestos Use

**DOI:** 10.3390/ijerph17020607

**Published:** 2020-01-17

**Authors:** Enrico Oddone, Jordy Bollon, Consuelo Rubina Nava, Marcella Bugani, Dario Consonni, Alessandro Marinaccio, Corrado Magnani, Francesco Barone-Adesi

**Affiliations:** 1 Department of Public Health, Experimental and Forensic Medicine, University of Pavia, 27100 Pavia, Italy; 2 Occupational Medicine Unit (UOOML), ICS Maugeri IRCCS, 27100 Pavia, Italy; 3 Department of Pharmaceutical Sciences, University of Eastern Piedmont, 28100 Novara, Italy; jbollon94@gmail.com (J.B.); francesco.baroneadesi@uniupo.it (F.B.-A.); 4 Department of Economics and Political Science, University of Aosta Valley, 11100 Aosta, Italy; c.nava@univda.it; 5 Occupational and Environmental Medicine, Epidemiology and Hygiene Department, Italian Workers’ Compensation Authority (INAIL), 00187 Rome, Italy; marcella.bugani@gmail.com (M.B.); a.marinaccio@inail.it (A.M.); 6 Epidemiology Unit, Fondazione IRCCS Ca’ Granda Ospedale Maggiore Policlinico, 20122 Milan, Italy; dario.consonni@unimi.it; 7 Unit of Medical Statistics and Cancer Epidemiology, Department of Translational Medicine, University of Eastern Piedmont, Novara, and CPO-Piedmont, 28100 Novara, Italy; corrado.magnani@med.uniupo.it

**Keywords:** pleural mesothelioma, epidemic, age–period–cohort, asbestos ban, trends

## Abstract

Even if the epidemic of malignant pleural mesothelioma (MPM) is still far from being over worldwide, the health effects of regulations banning asbestos can be evaluated in the countries that implemented them early. Estimates of MPM future burden can be useful to inform and support the implementation of anti-asbestos health policies all around the world. With this aim we described the trends of MPM deaths in Italy (1970–2014) and predicted the future number of cases in both sexes (2015–2039), with consideration of the national asbestos ban that was issued in 1992. The Italian National Statistical Institute (ISTAT) provided MPM mortality figures. Cases ranging from 25 to 89 years of age were included in the analysis. For each five-year period from 1970 to 2014, mortality rates were calculated and age–period–cohort Poisson models were used to predict future burden of MPM cases until 2039. During the period 1970–2014 a total number of 28,907 MPM deaths were observed. MPM deaths increased constantly over the study period, ranging from 1356 cases in 1970–1974 to 5844 cases in 2010–2014. The peak of MPM cases is expected to be reached in the period 2020–2024 (about 7000 cases). The decrease will be slow: about 26,000 MPM cases are expected to occur in Italy during the next 20 years (2020–2039). The MPM epidemic in Italy is far from being concluded despite the national ban implemented in 1992, and the peak is expected in 2020–2024, in both sexes. Our results are consistent with international literature.

## 1. Introduction

Malignant mesothelioma is one of the worst legacies of asbestos exposure, causing an estimated figure of 27,000 deaths per year worldwide [1,2]. As malignant pleural mesothelioma (MPM) is mainly caused by asbestos exposure [3], incidence and mortality for this disease are often used as a marker of previous exposures to asbestos. The observation of increasing trends of MPM occurrence provided a clear alarm regarding the impact of asbestos exposure [4], while the analyses of trends have been used to evaluate the effects of reducing asbestos exposure in the population during more recent periods [4,5,6]. Given the long time that usually elapses between first exposure to asbestos and development of MPM, several industrialized countries that banned asbestos a long time ago are now approaching the peak of MPM cases [7,8,9,10,11] or have reached it in the recent past [12,13,14]. In other countries, where the widespread use of asbestos was common until recently or is even still occurring [15], the increase in MPM incidence is expected to last for many decades [16].

Incidence of MPM in Italy rose constantly over the past decades, reaching one of highest rates in the world. [5,6,17,18,19]. Italy banned asbestos extraction, use, and commercialization in 1992 (Italian Law 257/92) and it is thus important to monitor the trend of MPM occurrence to detect the effects of such a ban. The large number of MPM cases expected in the next years is of particular concern due to the extremely poor prognosis of this condition and the current lack of effective therapeutic options [20].

In spite of several studies that previously tried to forecast the MPM burden [4,5,6,21], it is still unknown how long the MPM epidemic will last, both worldwide and in the countries that banned asbestos use [13,22,23]. In particular, it is not clear how slow will be the reduction of MPM mortality, once that peak will be reached. Up-to-date predictions of the future trend of MPM are thus necessary to inform public health interventions. The aim of the present paper is to describe the observed number of MPM deaths in Italy during the period 1970–2014 and to provide predictions of the number of deaths expected in the next decades. Predictions are provided separately by gender as the pattern of asbestos exposure is likely to be different, with occupational exposure playing a larger role in men and domestic/environmental exposure being more relevant for women, respectively [24]. 

## 2. Material and Methods

### 2.1. Data Source

Data were collected from the Italian National Statistical Institute (ISTAT). A specific death code for MPM was not available until 2003, when the tenth revision of the International Classification of Diseases was implemented in Italy. Thus, the annual number of MPM deaths in the period 1970–2002 was estimated by applying a correction factor to the number of deaths for pleural cancers (ICD codes: VIII revision: 163.0–163.0; IX revision: 163.0–163.9; X revision: C38.4, C45.0, C45.9) recorded in each year, as proposed by Ferrante and colleagues [25]. ISTAT mortality data were not available for 2004, thus missing data were calculated by interpolation of data from 2003 and 2005.The present analysis was restricted to cases aged between 25 and 89 years of age, as MPM is extremely rare before 25 and diagnosis less certain after 89 years of age. 

### 2.2. Statistical Analysis

We estimated MPM mortality rates for men and women for each five-year period from 1970 to 2014. Mortality rates by age at death, year of death, and birth cohort were plotted separately for men and women. We used Poisson age–period–cohort (APC) models to forecast MPM future trends [26]. Logarithm of person-years (py) was set as the offset in each model. Actual (1970–2014) and predicted (2015–2039) population data, stratified by year, gender, and age, were obtained by the National Institute of Statistics website (http://demo.istat.it/index_e.html). Gender-specific age, period, and cohort regression coefficients were then applied to population data to calculate projections of the numbers of cases of MPM and their 95% prediction intervals (PIs) for the years 2015–2039. PIs were computed using a bootstrap method proposed by Yang and colleagues [27].

A likelihood ratio (LR) test was used to compare the APC model with nested models (i.e., age–cohort and age–period models). An overall comparison among nested and not-nested models was also carried out using the Akaike Information Criterion (AIC). Data management and statistical analyses were performed with the APC R software package [28].

## 3. Results

During the period 1970–2014, a total number of 28,907 MPM deaths were observed, 20,245 (70.0%) among men and 8662 (30.0%) among women. MPM deaths increased constantly over the study period, ranging from 856 cases in 1970–1974 to 4275 cases in 2010–2014 among men, and from 500 cases in 1970–1974 to 1569 cases in 2010–2014 among women (Table 1.) The men-to-women ratio also increased, shifting from 1.73 in 1970–1974 to 2.72 in 2010–2014. MPM was uncommon under 45 years of age. During the study period, a constant decrease of the percentage of cases under 45 years of age was observed in both genders (5.02% to 0.58% and 7.06% to 0.57%, in men and women respectively). More than 80% of MPM cases in both sexes were aged 60 or older.

**Figure 1:**
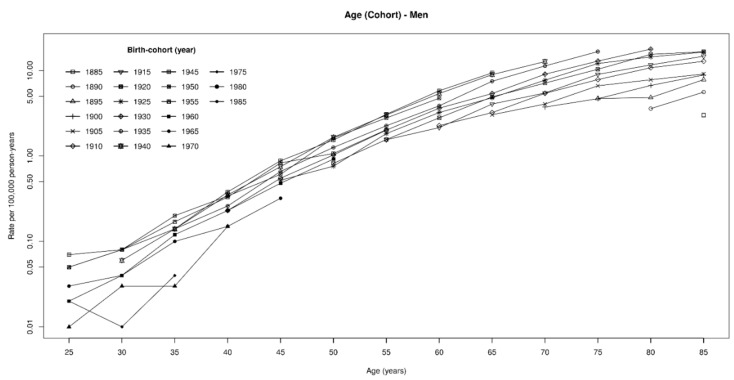

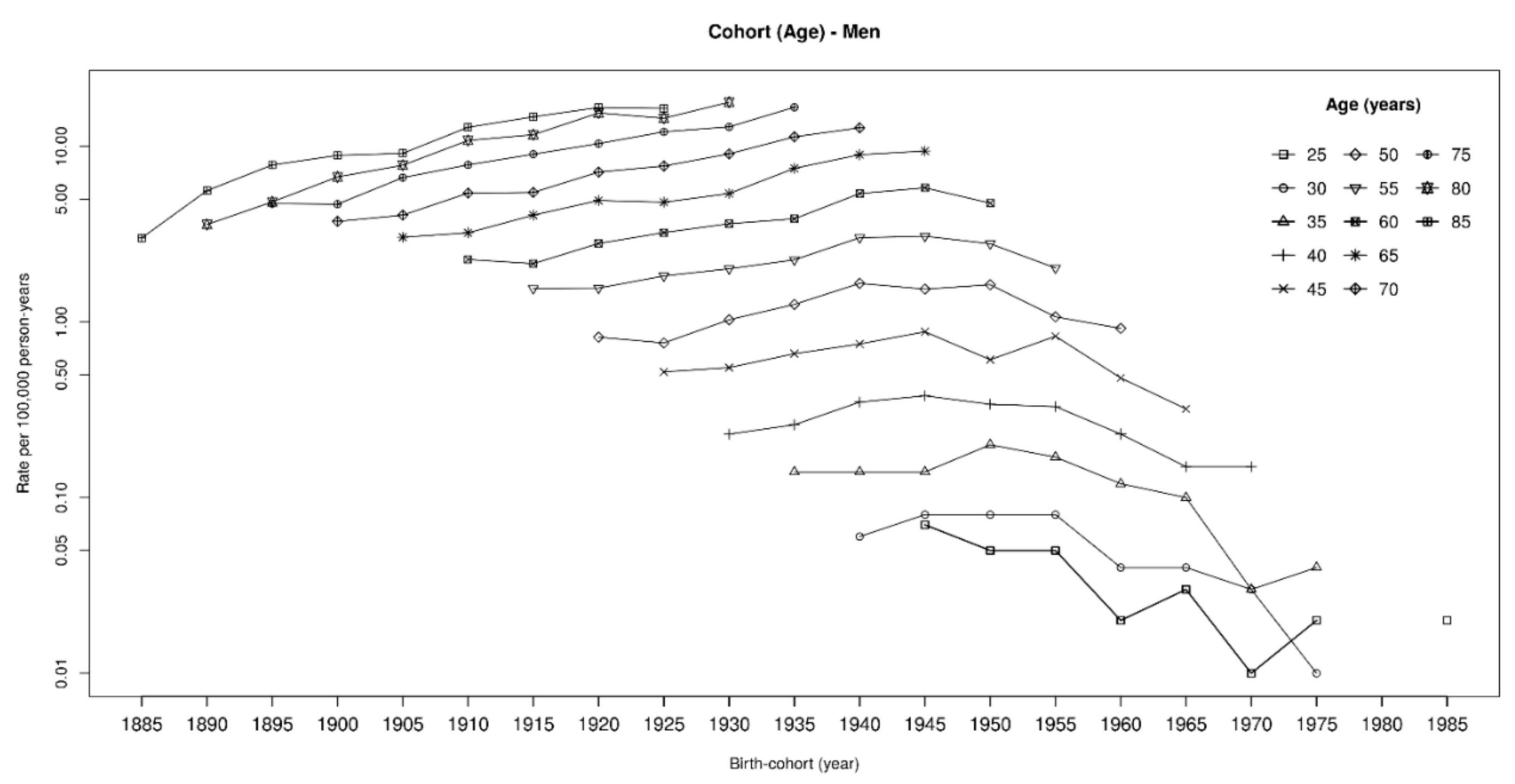
Malignant pleural mesothelioma death rates (×100,000) by age and birth cohort. Men, Italy, 1970–2014.

**Figure 2:**
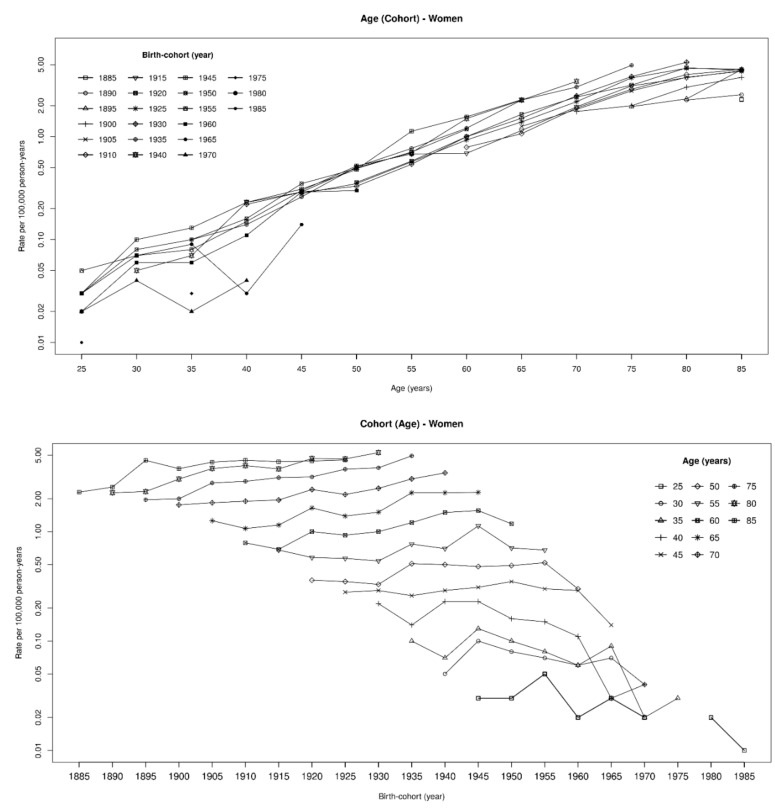
Malignant pleural mesothelioma death rates (×100,000) by age and birth cohort. Women, Italy, 1970–2014.

MPM rates increased constantly over time in both genders, reaching in 2014 a maximum of 3.99 per 100,000 person-years (py) in men and 1.34 per 100,000 py in women (Table 1). In addition, mortality rates increased by age within each birth cohort (Figure 1 and Figure 2 and Tables S1 and S2). Among women, rates were much lower than in men, being usually one third or less. In men, the highest rate (17.9 per 100,000) was observed in birth cohort 1930–1934 among people aged 80–84, while rates over 10 per 100,000 were observed in those born from 1910 to 1940 (Figure 1 and Table S1). The highest rate in women was observed in the birth cohort 1930–1934 (80–84 years of age, 5.3 per 100,000 py), and rates exceeding 4 per 100,000 py were observed in those born between 1895 and 1939 (Figure 2 and Table S2). Compared to previous years, a steep increase in MPM rates among those aged 60 or older was observed in both genders from 2000 onwards (Table 1 and Figure 1). In the period 1970–1999, on average, the yearly increase in MPM rates was about 4.5% and 1.2% in men and in women, respectively. These figures increased to 10.0% among men and 2.7% among women in 2000–2014.

The age–period–cohort (APC) model provided the best fit to the data. However, age–cohort (AC) provides a very similar fit, and predictions of APC and AC models were almost identical (Table 2, Figures S1 and S2).

**Figure 3:**
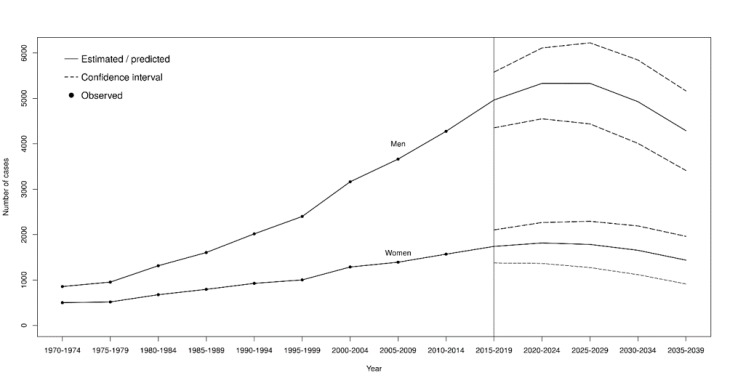
Observed and predicted number of cases of malignant pleural mesothelioma, with 95% predicted intervals. Age–period–cohort model, Italy, 1970–2039.

Observed (1970–2014) and predicted (2015–2039) numbers of MPM by gender are reported in Figure 3. About 19,500 and 6700 MPM cases are expected among men and women, respectively, by the next 20 years (2020–2039) in Italy. The peak of MPM cases is expected to occur during the 2020–2024 period for both genders, with about 5200 and 1800 MPM cases among men and women, respectively. The decrease following this peak will be slow: the predicted number of MPM cases in 2035–2039 will be about 80% of that expected during the peak (Figure 3). The decrease will be similar among the two genders, with a rather constant men-to-women ratio from 2015 to 2039, ranging between 2.89 and 2.98.

## 4. Discussion

This study evaluated the evolution of the MPM epidemic in Italy, providing both observed (1970–2014) and predicted mortality (2015–2039) figures, based on national mortality statistics provided by the Italian National Institute of Statistics (ISTAT). Our results suggest that the number of MPM cases is still increasing, with a predicted peak of more than 7000 deaths in the period 2020–2024.

Although the trend of the number of MPM deaths was constantly increasing along the 1970–2014 period, a further acceleration was observed since the end of the 1990s. This rise could be due to several factors, in particular to the large use of asbestos in Italy that occurred between the 1950s and the first half of the 1970s. Indeed, different authors showed that the risk of developing MPM is mainly related to exposure occurring three to four decades before [29]. The improvement of MPM diagnostic accuracy could also have played some role in this trend. Studied conducted in other countries reported a yearly 5% decrease of misdiagnosis of MPM starting from the 1990s [4,14,18,30].

According to our predictions, the peak in MPM cases is expected in the next few years (2020–2024), followed by a plateau and a slow decrease in the following decades. Notably, the predicted number of MPM cases from 2020 to 2039 (about 26,000) is very similar to the number of cases observed so far. This would imply that a substantial part of the MPM epidemic in Italy is still to come. However, it should be also considered that the models used for the present predictions could not completely capture the effects of the implementation of the asbestos ban in 1992. As the effects of such a ban are expected to become evident 30 to 40 years after its enforcement, the number of MPM cases that will be observed in the next few years will be relevant to provide accurate information on the future trends. As MPM is a disease that is more common among subjects aged >60 years, changes in the age structure of the population can substantially affect the predictions of future number of MPM as well. Thus, an increase of MPM cases could occur in an aging population even if age-specific incidence rates remain constant or decrease. A similar phenomenon has been recently observed in an age–period–cohort analysis of incidence data in the United States [14]. Finally, our predictions do not assume any future improvement in the prognosis of MPM, which is presently poor [31]. Any effective new treatment for MPM becoming available in the next years may reduce the future number of MPM deaths. 

Our study is one of the first to provide MPM forecasts for women. It is noteworthy that the men-to-women ratio showed a continuous increasing trend over 1970–2014 (from 1.73 in 1970–1994 to 2.72 in 2010–2014), with a larger increment after 1995. The largest absolute difference between cases in males and females (2706 cases) was observed in 2010–2014. However, the men-to-women ratio is predicted to remain rather constant in the future, suggesting that the shape of the future decreasing trend of MPM will be similar in both sexes. Also recent Italian incidence data depict a constant men-to-women ratio [32], thus contributing to corroborate our results. 

Our results are consistent with those from Italian studies conducted both at regional [19,33,34] and national [8] levels. In particular, previous predictions by Marinaccio et al. [8] for the male population, obtained by applying AC and APC models, suggested a peak of 890 annual deaths in the period 2020–2024. Analyzing incident cases of malignant mesothelioma (all sites) in the Lombardy region (northwest Italy) using an age–cohort model, Mensi et al. [19] found that the peak is expected around 2019. Conversely, the study by Girardi and colleagues in the Veneto region estimated a peak in the incidence of MPM cases in 2010 [33]. However, it should be also noted that, following the peak, this study predicted a plateau in MPM cases until 2026, thus partially overlapping with the national predictions.

Different studies suggest that national mortality data probably underestimate the actual number of mesotheliomas due to misdiagnosis, non-diagnosis, or lack of reporting [35,36]. Interestingly, extrapolations from the Global Burden of Disease (GBD) data suggest a substantially larger number of mesothelioma deaths in Italy than reported from the National Office of Statistics [37]. Although it is not clear how much of this apparent discrepancy is due to real misclassification, we note that the increasing temporal trend in MPM deaths that we observed is completely consistent with the one highlighted by GBD data [37]. In fact, the pattern of mesothelioma death rates by birth cohort and age observed in our data is similar to that estimated by other data sources, such as the same GBD database [37].

Worldwide, several studies tried to forecast the future burden of MPM. [4,5,6,21]. In Great Britain, Hodgson and colleagues predicted the MPM peak in the period 2011–2015 [7]. More recent studies substantially confirmed this prediction, placing the peak around 2016–2017 [9,38]. Several Nordic European countries show a pattern similar to Great Britain. In Denmark and the Netherlands, the peak of male MPM cases was predicted in 2015 and 2017, respectively [39,40]. Sweden is a notable exception, where the peak in MPM deaths was observed already in the 1990s [13], although a recent study seems to indicate a new increase in the number of deaths from mesothelioma after 2000 [37]. According to the most recent predictions, the number of MPM cases in Spain will increase at least until 2020 [41]. U.S. men have probably already reached the peak of MPM cases during the 2002–2007 period, although the MPM epidemic is predicted to last at least until 2042 [42]. In Canada, the peak of male MPM cases is predicted to be reached in 2020 [43]. In Brazil [44], the number of MPM cases is predicted to increase until 2026, while in Japan and South Korea the peak is expected around 2030 [16,45].

Differences in the period of maximum burden of MPM cases are largely determined by past national consumption of asbestos and the implementation of bans of its usage [29]. This inference is also supported by asbestos consumption models, where the gap between the peak of asbestos use and that of MPM cases is estimated to be about 30 to 40 years [7,8,41,43,44,45,46]. In our case, the Italian ban implemented in 1992 is probably starting just now to show its positive effects. Future studies will be useful to thoroughly evaluate the effect of the Italian asbestos ban and its efficacy in terms of MPM death reduction.

In general, the decrease of the number of MPM cases following the peak is generally predicted to be a slow process, with a large amount of cases expected after the peak. The case of Great Britain is a notable exception, given that all authors agreed on a rapid decrease of MPM cases following the peak [7,9,38]. The reason of the peculiar trend of Great Britain is presently unknown and warrants further investigations.

Our study predicts a slow decrease of MPM deaths following the peak. This picture is of particular concern for its clinical and preventive implications. Caring for MPM patients (irrespective of them being former asbestos workers or not) has to be viewed as a long-term program, which requires strong support for research for improving therapeutic options and for finding suitable and reliable markers allowing for an early diagnosis. Finally, compensation for occupational MPM has to be extended towards future decades, and remediation for environmental sources of exposure has to be strengthened to prevent further asbestos exposure and also to evaluate and manage the issue of asbestos in place, which at present is still an open question due to the lack of reliable data at the national level.

## 5. Conclusions 

Our results are consistent with the literature data highlighting that the MPM epidemic in Italy is still far from being concluded, despite the national ban implemented in 1992. Predictions of the future burden of disease could help to rationally program interventions devoted to the care of MPM patients, remediation of asbestos-contaminated sites, and compensation for occupational MPMs.

## Figures and Tables

**Table 1:** Pleural mesothelioma deaths and mortality rates (×100,000 person-years) by age and period, Italy, 1970–2014.

Age Group	Period									
	1970–1974	1975–1979	1980–1984	1985–1989	1990–1994	1995–1999	2000–2004	2005–2009	2010–2014	Total
**Male**										
25–29	6	5	5	2	3	1	2	.	2	**26**
	*0.07*	*0.05*	*0.05*	*0.02*	*0.03*	*0.01*	*0.02*	.	*0.02*	***0.03***
30–34	6	7	8	8	4	5	3	1	.	**42**
	*0.06*	*0.08*	*0.08*	*0.08*	*0.04*	*0.04*	*0.03*	*0.01*	.	***0.05***
35–39	13	13	13	19	16	12	12	4	5	**107**
	*0.14*	*0.14*	*0.14*	*0.20*	*0.17*	*0.12*	*0.10*	*0.03*	*0.04*	***0.12***
40–44	21	23	32	34	33	31	24	18	18	**234**
	*0.23*	*0.26*	*0.35*	*0.38*	*0.34*	*0.33*	*0.23*	*0.15*	*0.15*	***0.26***
45–49	46	49	58	68	77	58	77	50	38	**521**
	*0.52*	*0.55*	*0.66*	*0.75*	*0.88*	*0.61*	*0.83*	*0.48*	*0.32*	***0.61***
50–54	53	65	89	108	148	132	153	99	94	**941**
	*0.82*	*0.76*	*1.03*	*1.26*	*1.66*	*1.54*	*1.63*	*1.07*	*0.92*	***1.20***
55–59	106	96	148	166	187	260	256	256	185	**1660**
	*1.55*	*1.56*	*1.83*	*2.01*	*2.26*	*3.02*	*3.08*	*2.79*	*2.03*	***2.28***
60–64	155	136	159	241	282	303	441	466	423	**2606**
	*2.27*	*2.15*	*2.80*	*3.23*	*3.63*	*3.87*	*5.39*	*5.81*	*4.75*	***3.89***
65–69	159	190	225	248	324	380	541	691	715	**3473**
	*3.04*	*3.22*	*4.06*	*4.92*	*4.81*	*5.39*	*7.50*	*8.97*	*9.41*	***5.99***
70–74	133	167	255	250	306	445	556	734	895	**3741**
	*3.74*	*4.06*	*5.42*	*5.47*	*7.14*	*7.73*	*9.07*	*11.34*	*12.76*	***8.03***
75–79	100	115	188	271	316	348	554	655	915	**3462**
	*4.72*	*4.69*	*6.64*	*7.85*	*9.03*	*10.37*	*12.11*	*12.92*	*16.71*	***10.54***
80–84	43	59	93	133	243	273	358	471	670	**2343**
	*3.59*	*4.83*	*6.70*	*7.81*	*10.81*	*11.66*	*15.50*	*14.46*	*17.86*	***12.07***
85–89	15	29	41	57	77	151	186	218	315	**1089**
	*3.01*	*5.60*	*7.84*	*8.89*	*9.13*	*12.85*	*14.74*	*15.26*	*16.65*	***12.54***
**Total**	**856**	**954**	**1314**	**1605**	**2016**	**2399**	**3163**	**3663**	**4275**	**20245**
	***1.09***	***1.17***	***1.57***	***1.84***	***2.18***	***2.47***	***3.14***	***3.51***	***3.99***	
**Female**										
25–29	3	3	5	2	3	2	.	2	1	**21**
	*0.03*	*0.03*	*0.05*	*0.02*	*0.03*	*0.02*	.	*0.02*	*0.01*	***0.02***
30–34	5	9	8	7	6	8	4	.	.	**47**
	*0.05*	*0.10*	*0.08*	*0.07*	*0.06*	*0.07*	*0.04*	.	.	***0.05***
35–39	9	7	12	10	8	6	10	2	3	**67**
	*0.10*	*0.07*	*0.13*	*0.10*	*0.08*	*0.06*	*0.09*	*0.02*	*0.03*	***0.07***
40–44	21	13	22	21	16	14	11	3	5	**126**
	*0.22*	*0.14*	*0.23*	*0.23*	*0.16*	*0.15*	*0.11*	*0.03*	*0.04*	***0.14***
45–49	26	27	24	27	28	34	29	30	17	**242**
	*0.28*	*0.29*	*0.26*	*0.29*	*0.31*	*0.35*	*0.30*	*0.29*	*0.14*	***0.28***
50–54	26	32	30	46	46	43	47	50	32	**352**
	*0.36*	*0.35*	*0.33*	*0.51*	*0.50*	*0.48*	*0.49*	*0.52*	*0.30*	***0.43***
55–59	51	41	51	48	68	64	99	68	66	**556**
	*0.68*	*0.58*	*0.57*	*0.54*	*0.77*	*0.70*	*1.13*	*0.71*	*0.68*	***0.71***
60–64	61	50	68	81	87	105	134	135	113	**834**
	*0.79*	*0.69*	*1.00*	*0.93*	*1.00*	*1.21*	*1.50*	*1.56*	*1.18*	***1.11***
65–69	80	76	79	105	115	126	189	197	193	**1160**
	*1.26*	*1.07*	*1.15*	*1.65*	*1.39*	*1.51*	*2.27*	*2.27*	*2.29*	***1.69***
70–74	87	102	120	120	142	168	195	241	286	**1461**
	*1.76*	*1.84*	*1.90*	*1.95*	*2.43*	*2.19*	*2.49*	*3.04*	*3.45*	***2.41***
75–79	67	78	124	152	165	163	255	273	358	**1635**
	*1.96*	*2.00*	*2.79*	*2.89*	*3.12*	*3.17*	*3.73*	*3.84*	*4.94*	***3.36***
80–84	44	53	80	120	160	155	195	263	315	**1385**
	*2.27*	*2.33*	*3.03*	*3.78*	*4.02*	*3.75*	*4.67*	*4.63*	*5.29*	***4.08***
85–89	20	26	53	55	82	114	118	128	180	**776**
	*2.30*	*2.56*	*4.49*	*3.77*	*4.33*	*4.50*	*4.37*	*4.43*	*4.53*	***4.19***
**Total**	**500**	**517**	**676**	**794**	**926**	**1002**	**1286**	**1392**	**1569**	**8662**
	***0.58***	***0.57***	***0.72***	***0.82***	***0.91***	***0.94***	***1.16***	***1.22***	***1.34***	

**Table 2:** Mortality rates for malignant pleural mesothelioma. Age–Period–Cohort analysis. Comparisons of different models.

Model	Men				Women			
	AIC	LR Statistic	*p* Value	DF	AIC	LR Statistic	*p* Value	DF
Age–period–cohort	834.132	NA	NA	77	783.076	NA	NA	77
Period–cohort	1613.485	801.353	<0.001	88	925.738	164.661	<0.001	88
Age–cohort	841.691	21.559	<0.003	84	783.557	14.481	0.043	84
Age–period	1418.905	622.773	<0.001	96	967.225	222.148	<0.001	96
Cohort	21,078.599	20,282.467	<0.001	96	7049.860	6211.774	<0.001	96
Period	33,583.124	32,810.992	<0.001	108	12,790.662	11,997.016	<0.001	108
Age	3318.674	2538.542	<0.001	104	1207.498	505.611	<0.001	104

AIC: Akaike Information Criterion. LR: Likelihood ratio. NA: not applicable.

## Supplementary Materials

The following are available online at https://www.mdpi.com/1660-4601/17/2/607/s1, Figure S1: Predictions by AC and APC models for men, Italy. 1970–2039, Figure S2: Predictions by AC and APC models for women, Italy. 1970–2039; Table S1: Mortality rates of malignant pleural mesothelioma (x 100 000 person-years) in men by birth cohort and age at diagnosis. Italy. 1970–2014. Table S2: Mortality rates of malignant pleural mesothelioma (x 100 000 person-years) in women by birth cohort and age at diagnosis. Italy. 1970–2014. Table S3: APC predictions and detailed data for 5-year categories.
