# Supplementary material for: Predictions of Mortality from Pleural Mesothelioma in Italy After the Ban of Asbestos Use
Source: Int J Environ Res Public Health. 2020 Jan 17;17(2):607. doi: 10.3390/ijerph17020607 (PMC7013387; doi:10.3390/ijerph17020607)
Supplement: Supplementary file 1 [file ijerph-17-00607-s001.zip › ijerph-686788-supplementary/Table S3.docx]

The underlying Poisson assumption of the APC models used in the analysis explains the differences in the width of the predicted intervals between men and women. In Poisson models, the variance increases with the mean number of events. As the number of cases was about two times larger in men than in women, predicted intervals (PI) of men are larger than those of women. Please also note that, on the relative scale, predicted intervals are smaller for men than for women. This can be evaluated through the coefficient of variation, reported in the table thereafter.

In what follows, we report the bootstrap algorithm used to determine PI and detailed data for 5-year categories. This information has now been added in the paper as supplementary material.

1. Fit the APC model and retain the fitted link values *μ̂_i_* and the residual *ε̂_i_ = μ_i_ – μ̂_i_ = log(y_i_) – μ̂_i_*,

*i = 1, 2, ..., n*, where *n* indicates the number of observations or the number of age groups times the number of period groups p.

2. For each pair *x_i_*, *μ_i_* in which x_i_ is the explanatory variable, add a randomly resampled residual *ε̂_j_* to the link variable *μ_i_*. In other words, create the synthetic link and response variables *μ^*^_i_ = μ̂_i_ + ε̂_j_* and *y_i_^*^ = exp(μ^*^_i_)* where *j* is selected randomly from the list (*1, ..., n*) for every *i*.

3. Refit the model using the fictitious response variables *y_i_^*^*, make forecasting based on the synthetic *ŷ_i_^*^* as from the prediction procedure, and retain the predicted *ŷ_i_^*^*.

4. Repeat Steps 2 and 3 a large number of times (1,000 times here) to obtain (1,000) samples of *ŷ_i_** and derive the related mean, standard errors, and PIs.

Taking advantage of the above algorithm, we estimate the standard errors of the future MPM cases by computing the standard deviation of 1,000 predicted *ŷ_i_**. Below a table with further details.

Table S3 – APC predictions and detailed data for 5-year categories.

|  |  | 2015-2019 | 2020-2024 | 2025-2029 | 2030-2034 | 2035-2039 |
| --- | --- | --- | --- | --- | --- | --- |
| Male | APC predictions | 4965 | 5331 | 5331 | 4928 | 4288 |
|  | Bootstrap SE | 307 | 395 | 453 | 470 | 452 |
|  | PI width ^^[[1]](#footnote-1)^^ | 1203 | 1548 | 1776 | 1842 | 1772 |
|  | Coefficient of Variation (CV)^^[[2]](#footnote-2)^^ | 0.062 | 0.074 | 0.085 | 0.095 | 0.105 |
| Female | APC predictions | 1740 | 1815 | 1783 | 1654 | 1438 |
|  | Bootstrap SE | 187 | 231 | 264 | 280 | 269 |
|  | PI width ^1^ | 733 | 906 | 1035 | 1098 | 1054 |
|  | Coefficient of Variation (CV)^2^ | 0.107 | 0.127 | 0.148 | 0.169 | 0.187 |

^1^ Width = 2 * Bootstrap SE * 1.96.

^2^ CV = Bootstrap SE / APC predictions.

1. [↑](#footnote-ref-1)
2. [↑](#footnote-ref-2)
