# Supplementary material for: Predictions of Mortality from Pleural Mesothelioma in Italy After the Ban of Asbestos Use
Source: Int J Environ Res Public Health. 2020 Jan 17;17(2):607. doi: 10.3390/ijerph17020607 (PMC7013387; doi:10.3390/ijerph17020607)
Supplement: Supplementary file 1 [file ijerph-17-00607-s001.zip › ijerph-686788-supplementary/Table S1 - post rev j.docx]

**Table S1** – Mortality rates of malignant pleural mesothelioma (x 100 000 py) in men by birth cohort and age at diagnosis. Italy. 1970-2014.

|  | **1885** | **1890** | **1895** | **1900** | **1905** | **1910** | **1915** | **1920** | **1925** | **1930** | **1935** | **1940** | **1945** | **1950** | **1955** | **1960** | **1965** | **1970** | **1975** | **1980** | **1985** |
| --- | --- | --- | --- | --- | --- | --- | --- | --- | --- | --- | --- | --- | --- | --- | --- | --- | --- | --- | --- | --- | --- |
| **25-29** | NA | NA | NA | NA | NA | NA | NA | NA | NA | NA | NA | NA | 0.066 | 0.050 | 0.052 | 0.019 | 0.026 | 0.009 | 0.019 | 0.000 | 0.024 |
| **30-34** | NA | NA | NA | NA | NA | NA | NA | NA | NA | NA | NA | 0.063 | 0.078 | 0.081 | 0.084 | 0.039 | 0.043 | 0.026 | 0.009 | 0.000 | NA |
| **35-39** | NA | NA | NA | NA | NA | NA | NA | NA | NA | NA | 0.143 | 0.139 | 0.145 | 0.195 | 0.168 | 0.116 | 0.104 | 0.034 | 0.044 | NA | NA |
| **40-44** | NA | NA | NA | NA | NA | NA | NA | NA | NA | 0.230 | 0.256 | 0.347 | 0.383 | 0.340 | 0.328 | 0.234 | 0.153 | 0.150 | NA | NA | NA |
| **45-49** | NA | NA | NA | NA | NA | NA | NA | NA | 0.523 | 0.547 | 0.658 | 0.750 | 0.877 | 0.606 | 0.825 | 0.484 | 0.322 | NA | NA | NA | NA |
| **50-54** | NA | NA | NA | NA | NA | NA | NA | 0.817 | 0.764 | 1.027 | 1.261 | 1.664 | 1.536 | 1.634 | 1.066 | 0.915 | NA | NA | NA | NA | NA |
| **55-59** | NA | NA | NA | NA | NA | NA | 1.547 | 1.559 | 1.832 | 2.011 | 2.261 | 3.023 | 3.081 | 2.785 | 2.025 | NA | NA | NA | NA | NA | NA |
| **60-64** | NA | NA | NA | NA | NA | 2.271 | 2.154 | 2.797 | 3.229 | 3.635 | 3.874 | 5.391 | 5.808 | 4.755 | NA | NA | NA | NA | NA | NA | NA |
| **65-69** | NA | NA | NA | NA | 3.042 | 3.223 | 4.056 | 4.920 | 4.812 | 5.392 | 7.501 | 8.973 | 9.408 | NA | NA | NA | NA | NA | NA | NA | NA |
| **70-74** | NA | NA | NA | 3.739 | 4.064 | 5.415 | 5.471 | 7.142 | 7.735 | 9.069 | 11.345 | 12.764 | NA | NA | NA | NA | NA | NA | NA | NA | NA |
| **75-79** | NA | NA | 4.722 | 4.686 | 6.635 | 7.852 | 9.035 | 10.366 | 12.112 | 12.916 | 16.706 | NA | NA | NA | NA | NA | NA | NA | NA | NA | NA |
| **80-84** | NA | 3.593 | 4.830 | 6.698 | 7.813 | 10.813 | 11.660 | 15.497 | 14.463 | 17.864 | NA | NA | NA | NA | NA | NA | NA | NA | NA | NA | NA |
| **85-89** | 3.013 | 5.599 | 7.841 | 8.889 | 9.134 | 12.850 | 14.738 | 16.650 | 16.469 | NA | NA | NA | NA | NA | NA | NA | NA | NA | NA | NA | NA |
