# Supplementary material for: Predictions of Mortality from Pleural Mesothelioma in Italy After the Ban of Asbestos Use
Source: Int J Environ Res Public Health. 2020 Jan 17;17(2):607. doi: 10.3390/ijerph17020607 (PMC7013387; doi:10.3390/ijerph17020607)
Supplement: Supplementary file 1 [file ijerph-17-00607-s001.zip › ijerph-686788-supplementary/Table S2 - post rev j.docx]

**Table S2** – Mortality rates of malignant pleural mesothelioma (x 100 000 py) in women by birth cohort and age at diagnosis. Italy. 1970-2014.

|  | **1885** | **1890** | **1895** | **1900** | **1905** | **1910** | **1915** | **1920** | **1925** | **1930** | **1935** | **1940** | **1945** | **1950** | **1955** | **1960** | **1965** | **1970** | **1975** | **1980** | **1985** |
| --- | --- | --- | --- | --- | --- | --- | --- | --- | --- | --- | --- | --- | --- | --- | --- | --- | --- | --- | --- | --- | --- |
| 25-29 | NA | NA | NA | NA | NA | NA | NA | NA | NA | NA | NA | NA | 0,033 | 0,03 | 0,052 | 0,019 | 0,026 | 0,018 | 0 | 0,022 | 0,012 |
| 30-34 | NA | NA | NA | NA | NA | NA | NA | NA | NA | NA | NA | 0,052 | 0,099 | 0,081 | 0,073 | 0,058 | 0,07 | 0,035 | 0 | 0 | NA |
| 35-39 | NA | NA | NA | NA | NA | NA | NA | NA | NA | NA | 0,097 | 0,073 | 0,132 | 0,102 | 0,084 | 0,058 | 0,087 | 0,017 | 0,026 | NA | NA |
| 40-44 | NA | NA | NA | NA | NA | NA | NA | NA | NA | 0,224 | 0,141 | 0,233 | 0,233 | 0,163 | 0,147 | 0,107 | 0,025 | 0,041 | NA | NA | NA |
| 45-49 | NA | NA | NA | NA | NA | NA | NA | NA | 0,279 | 0,291 | 0,262 | 0,289 | 0,312 | 0,349 | 0,305 | 0,285 | 0,141 | NA | NA | NA | NA |
| 50-54 | NA | NA | NA | NA | NA | NA | NA | 0,363 | 0,348 | 0,327 | 0,511 | 0,497 | 0,485 | 0,487 | 0,52 | 0,298 | NA | NA | NA | NA | NA |
| 55-59 | NA | NA | NA | NA | NA | NA | 0,676 | 0,585 | 0,566 | 0,536 | 0,768 | 0,702 | 1,133 | 0,706 | 0,682 | NA | NA | NA | NA | NA | NA |
| 60-64 | NA | NA | NA | NA | NA | 0,794 | 0,687 | 1,004 | 0,934 | 0,999 | 1,215 | 1,503 | 1,565 | 1,184 | NA | NA | NA | NA | NA | NA | NA |
| 65-69 | NA | NA | NA | NA | 1,262 | 1,066 | 1,152 | 1,65 | 1,391 | 1,508 | 2,267 | 2,271 | 2,292 | NA | NA | NA | NA | NA | NA | NA | NA |
| 70-74 | NA | NA | NA | 1,763 | 1,842 | 1,896 | 1,946 | 2,426 | 2,186 | 2,487 | 3,043 | 3,453 | NA | NA | NA | NA | NA | NA | NA | NA | NA |
| 75-79 | NA | NA | 1,963 | 2,000 | 2,792 | 2,886 | 3,125 | 3,166 | 3,727 | 3,84 | 4,943 | NA | NA | NA | NA | NA | NA | NA | NA | NA | NA |
| 80-84 | NA | 2,271 | 2,332 | 3,027 | 3,776 | 4,024 | 3,751 | 4,672 | 4,632 | 5,286 | NA | NA | NA | NA | NA | NA | NA | NA | NA | NA | NA |
| 85-89 | 2,298 | 2,56 | 4,493 | 3,769 | 4,334 | 4,496 | 4,371 | 4,425 | 4,528 | NA | NA | NA | NA | NA | NA | NA | NA | NA | NA | NA | NA |
