# Supplementary figures and images for: Predictions of Mortality from Pleural Mesothelioma in Italy After the Ban of Asbestos Use
Source: Int J Environ Res Public Health. 2020 Jan 17;17(2):607. doi: 10.3390/ijerph17020607 (PMC7013387; doi:10.3390/ijerph17020607)

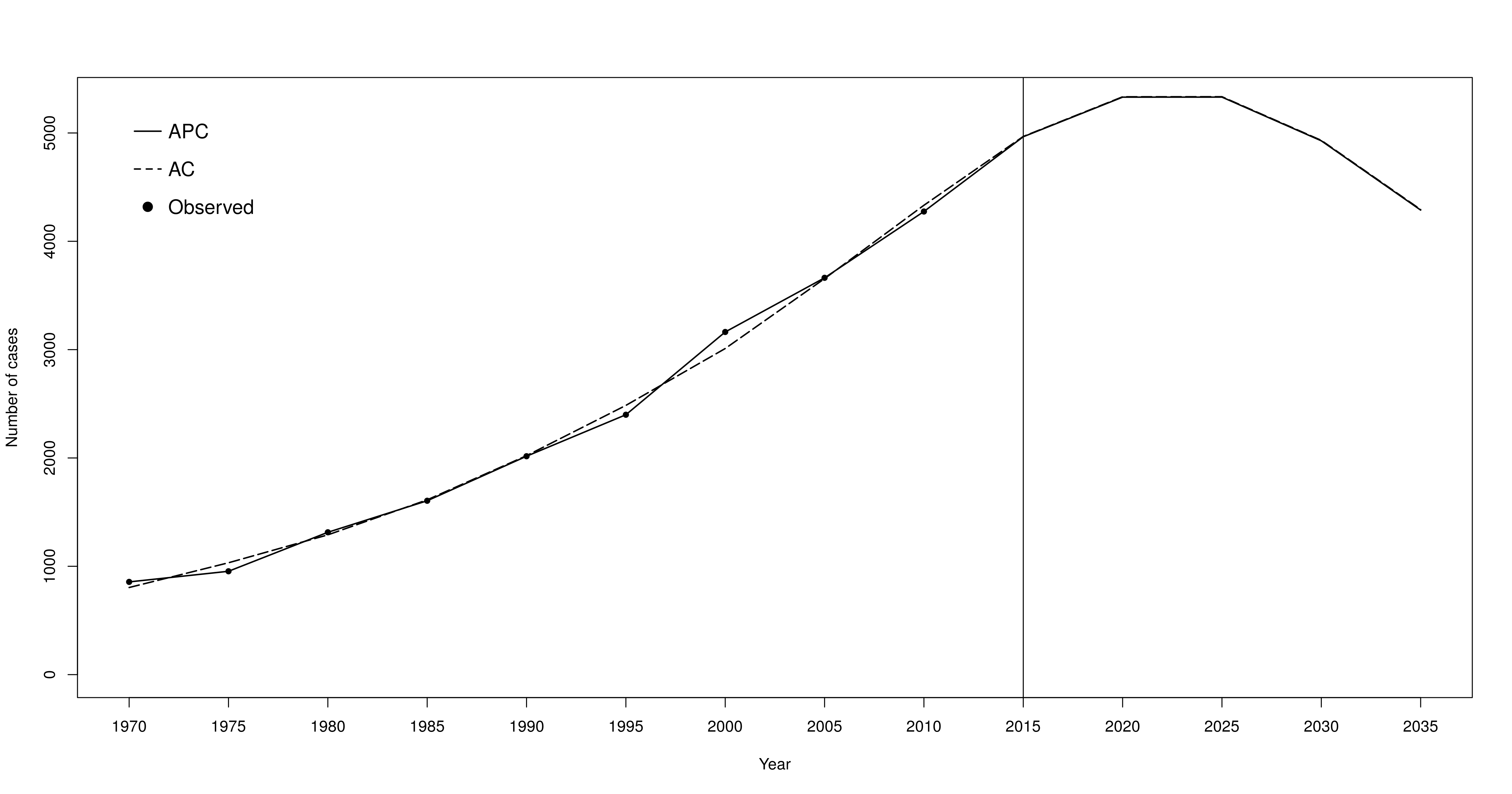

Supplement: Supplementary file 1 [file ijerph-17-00607-s001.zip › ijerph-686788-supplementary/S1.tiff]

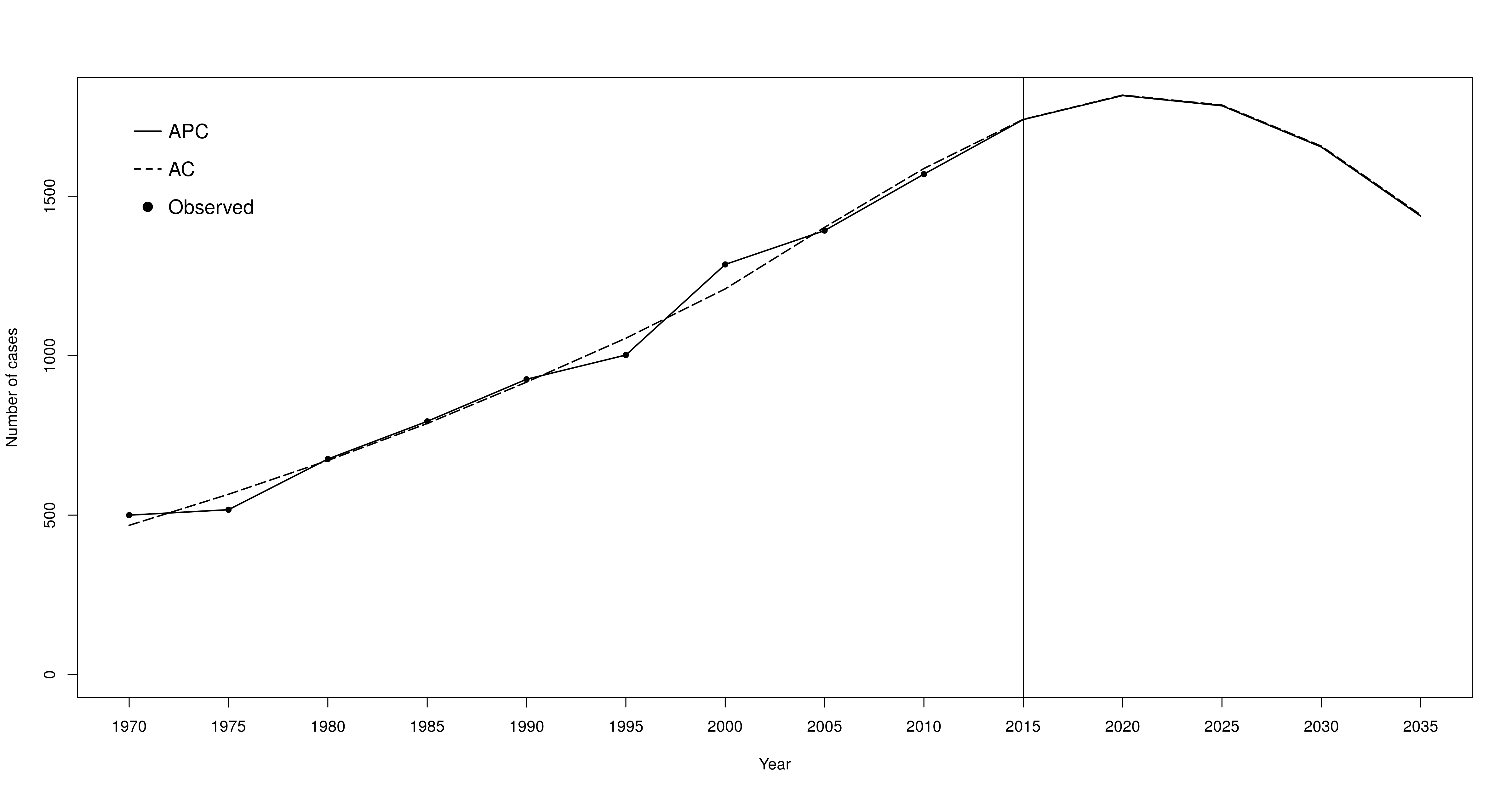

Supplement: Supplementary file 1 [file ijerph-17-00607-s001.zip › ijerph-686788-supplementary/S2.tiff]
